# Validation of Transition Readiness Assessment Questionaire in Turkish Adolescents with Diabetes

**DOI:** 10.4274/balkanmedj.2016.1415

**Published:** 2018-01-20

**Authors:** Evrim Kızıler, Dilek Yıldız, Berna Eren Fidancı

**Affiliations:** 1 Clinic of Pediatrics, Yıldırım Beyazıt University School of Medicine, Ankara, Turkey; 2 Department of Pediatric Nursing, University of Health Sciences, Gülhane School of Nursing, Ankara, Turkey

**Keywords:** Transition readiness, self-assessment questionnaire, youth with special health care needs, diabetes mellitus, transitional care

## Abstract

**Background:**

Today, more than 90% of adolescents with chronic conditions survive into adulthood and move from pediatric care to adult care for the management of their chronic illness. It is important to grant autonomy and ensure that adolescents/young adults are ready to use the adult health care system prior to the transfer of care. However, the lack of a transition readiness assessment tool that is validated, patient-centered, and appropriate to developmental differences in pediatric care is a major obstacle for the transition of care from pediatric services to adult services.

**Aims:**

This study examined the validity and reliability of the Turkish version of the Transition Readiness Assessment Questionnaire, which assesses the readiness for transition from pediatric to adult health care for adolescents/young adults with diabetes mellitus.

**Study Design:**

Methodological study.

**Methods:**

Participants were 109 adolescents/young adults with type 1 diabetes mellitus aged 14-21 years. After permission was obtained to adapt the Transition Readiness Assessment Questionnaire, the Turkish Transition Readiness Assessment Questionnaire and self-care scale were administered to participants through face-to-face interviews at two pediatric endocrinology clinics. Validity was evaluated by exploratory and confirmatory factor analysis and content-scope validity assessment; reliability was evaluated by item-total score correlation and continuity methods. Internal reliability was assessed by Cronbach’s alpha coefficient and criterion validity assessment.

**Results:**

The item analysis, exploratory factor analysis, and confirmatory factor analysis identified five basic dimensions, with high internal consistency (0.89-0.75). The ratio χ2/df and other conformity indices were a good fit to the data. The correlation coefficient in the analyses of test-retest scores was 0.86 for the total scale (p<0.05), and the Cronbach’s alpha coefficient was 0.88 for overall scale.

**Conclusion:**

The Turkish Transition Readiness Assessment Questionnaire is a valid and reliable measure of the transition readiness of adolescents/young adults with diabetes mellitus in Turkey. The Transition Readiness Assessment Questionnaire assesses the self-management abilities and health care transition knowledge of adolescents/young adults with diabetes mellitus who need special health care. It can also serve as a guide for health care professionals in detecting the educational fields that are necessary for acquiring self-management and self-care abilities.

Diabetes mellitus, a leading childhood disease, is a chronic disease that affects the quality of life of the affected child and of his/her parents because of its complicated and lifelong treatments. Diet regimens, exercise programs, and insulin treatments are standard, and certain developmental periods require additional compensatory treatments (1,2). A smooth transition from pediatric to adult care is necessary because the disease lasts a lifetime and adolescents enter adulthood with the disease (3,4,5,6,7,8,9,10).

In fact, more than 90% of adolescents who have chronic diseases reach adulthood with their diseases (11); 46% of these patients have difficulties and 11%-41% of them give up their diabetes mellitus care and control because they are unprepared for the adult health care system (2,12). The differences in the care and approaches between pediatric and adult units and the lack of care planning and provider support make the transition particularly challenging for adolescents/young adults (AYA) (1,2,9,13,14). Several studies investigating the transition experiences of patients with diabetes mellitus have reported that the young adults had the impression that they were considered less important because of the differences in approach between pediatric and adult units (1,15).

The transition is characterized as a period in which the adolescents obtain self management of the adult health care system, the disease, their lives, and become ready to take responsibility for their own health care (16,17). During this period, adolescents, who are typically under the control of their parents and pediatric health professionals during pediatric care, are expected to autonomously handle issues related to self care, health care decision-making, and self management that will prepare them to take more responsibility of their health (7,12,13,16,18,19,20,21). The American Academy of Pediatrics recommends that the transition should be initiated between 12 and 14 years of age; however, there is no consensus about when the maintenance and finishing phases of the process should be fulfilled because physical and psychological development do not necessarily correlate with the individual’s chronological age (20). Therefore, for the best transition timing, transition guidelines recommend that the adolescent’s transition readiness and ability to self manage and negotiate the adult health care system should be evaluated routinely and that the transition time should be set in accordance with the individual’s readiness (13,18,20,22). Nevertheless, in pediatric care, the lack of a validated and patient-centered scale to assess transition readiness and skills, such as the ability to make an appointment and understand medication instructions, is considered a major obstacle to the transition (18,20,23). It was thought that the adaptation of the Transition Readiness Assessment Questionnaire (TRAQ) to suit the Turkish population would be useful in assessing the transition readiness of adolescents and young adults in Turkey. Transition readiness is not all that may be assessed; the health care transition (HCT) knowledge and skills needed to transition may also be evaluated with this scale. In addition, this scale could guide health care professionals in preparing adolescents for the transition.

## MATERIALS AND METHODS

### Ethics

At the beginning of the study, approval was obtained for the use of the scales from the developers of the questionnaires. Ethics committee approval was granted for this study from local ethics committees. Ethical approval for the study was taken from Gülhane Military Medical Academy (E.C. Number: 50687469-1491-164-15/1648-4-289) and Ankara Childen and Oncology Hematology Training and Research Hospital (E.C. Number: 13.05.2015/18) local ethic committees. Written informed consent was obtained from the adolescents who participated in this study and their parents.

### Design and patient population

This study was planned and applied as a methodological study. The study was conducted in the pediatric endocrinology clinics of two education and research hospitals in Ankara between June and September 2015. The research sample comprised 109 volunteer, randomly selected adolescents and young adults with type 1 diabetes mellitus aged 14-21 years. The inclusion criteria were as follows: (a) being an adolescent/young adult with a diagnosis of diabetes mellitus for at least 1 year; (b) ability to read and understand Turkish; (c) willingness to participate in the study; (d) age between 14 and 21 years; and (e) being able to communicate. To adapt a scale for use in another culture, at least 5-10 times the item number is required (24). The sample size for the validity and reliability study of the TRAQ, which has 20 items, was calculated as (20 items × 5 Likert preference=100) (24).

### Measures and data

After explaining the study and required information, the sociodemographic form (the Turkish TRAQ) and self-care scale were administered to patients in a face-to-face interview. Completion of the questionnaires took approximately 30 min. Fifty-four adolescents with diabetes mellitus who answered the scale in the first application were requested to complete the scale again after a 3-week interval.

The Transition Readiness Assessment Questionnaire: The TRAQ examines the readiness for HCT of AYA with chronic diseases in 20 items and five dimensions, which are (a) managing medications, (b) keeping appointment, (c) tracking health issues, (d) talking with providers, and (e) managing daily activities. The scale is based on the stages of change and transtheoretical model that focuses on the impact of social and biological variables on behavior and decision-making skills of the individual (Table 1) (25). The TRAQ was developed; validity and reliability studies were conducted by Sawicki et al. (18), and redefined, shortened validity, and reliability studies were conducted by Wood et al. (12). The TRAQ is a 5-point Likert scale that includes 20 items that include a word intended to describe different qualities of skills for managing the disease (13). Statements in the questionnaire are assigned a score ranging from 1 (No, I don’t know how to do this) to 5 (Yes, I always do this when I need to), with a total possible score range of 20-100. Higher scores indicate increased readiness for HCT. Completing the scale takes approximately 15 min.

Self-care scale: The self-care agency scale, which was developed by Kearney and Fleischer, was validated for the Turkish population by Nahcivan (26). The self-care scale is a 5-point Likert-type scale that includes 35 items and evaluates the self-care ability of the patient. Each item of the scale is assigned a score between 0 (Never) and 4 (Always), with a total possible score of 35-140. Higher scores indicate increased self-care capacity (27).

### Equivalence of language and content validity of the scale

After obtaining permission to adapt the TRAQ into Turkish, the translation and back-translation method was used with expert opinions to determine content validity. First, three English-language experts and two native Turkish researchers who speak English fluently independently translated the original scale into Turkish. The translations were combined and prepared as a single text that was then retranslated into English by two other English-language experts. Next, the expert opinions of two nursing academicians experienced in care transition and research methods, a biostatistician, and a pediatric endocrinologist were incorporated. The scale was finalized; no more changes were made, and all 20 items in the questionnaire were included in the final form of the Turkish TRAQ.

### Statistical analysis

Statistical Package for the Social Sciences version 15.0 (SPSS Inc.; Chicago, IL, USA) and Statistical Package for the Social Sciences version 21.0-AMOS (SPSS Inc.; Chicago, IL, USA) were used for statistical analyses. Descriptive statistics were presented as frequencies and percentages for categorical variables and means, standard deviations, and ranges for continuous variables. Both exploratory factor analysis (EFA) and varimax rotation were performed to examine the scale’s construct validity. The Kaiser–Meyer–Olkin (KMO) coefficient and Bartlett’s test were used to evaluate the appropriateness of the sample size. Principal component analysis was used for factor extraction. Factors that had eigenvalues of more than 1 and that were located up to the place that the scree plot flattened were extracted. The EFA results were retested in the confirmatory factor analysis (CFA). The self-care scale was used for criterion validity assessment of the scale. Cronbach’s alpha coefficients, item-total subscale correlations, and repeatability of the scale were used to establish the internal consistency of the scale.

## RESULTS

Data were collected from 109 AYA to assess the validity and reliability of the TRAQ, which determines the readiness of transition from pediatric care to adult care of adolescents with chronic diseases and guides health care professionals in readying adolescents for transition–in the Turkish language.

### Sample characteristics

Participants from the two hospitals had similar demographic characteristics; 53.2% of the adolescents were male (n=58). The average age was 15.28±1.44 years; the mean age at diagnosis was 10.47 years (range, 2.0-16.0 years) and the average disease duration was 4.8 years (range, 1.0-15.0 years). Majority of the participants (56.9%, n=62) did not record fasting blood glucose results regularly, 52.3% (n=57) participants took their medications regularly without anyone’s reminder or warning, and 82.6% participants (n=90) reported that their lifestyle and activity level was the same as their peers. Moreover, 26.6% of the participants (n=29) contacted their physician themselves, and 30% (n=33) reported that the parents of the participants got in contact with the physician on their behalf.

### Validity of the Transition Readiness Assessment Questionnaire

***Exploratory factor analysis ***

EFA was used to determine the variables of the scale, and a solution similar with the factor structure in the original scale was found out. Suitability of the data for factor analysis was investigated, and the KMO value, which indicates the sample’s sensitivity to the application, was 0.734. This result indicates that the sample size was sufficient for factor analysis. After the evaluation of the Barlett’s test results (p<0.01), it was concluded that there was enough association between variables to perform factor analysis (28,29,30). Principal component and orthogonal varimax rotation technique were used to analyze factor loads, and they were found to be between 0.47 and 0.83. Items that had an eigenvalue higher than 1 were considered as factors (29,30). As a result of the EFA, the TRAQ was formed as a quintet factor and the variance defined by these five basic subdimensions was 74% (Table 2).

***Confirmatory factor analysis***

**Figure 1:**
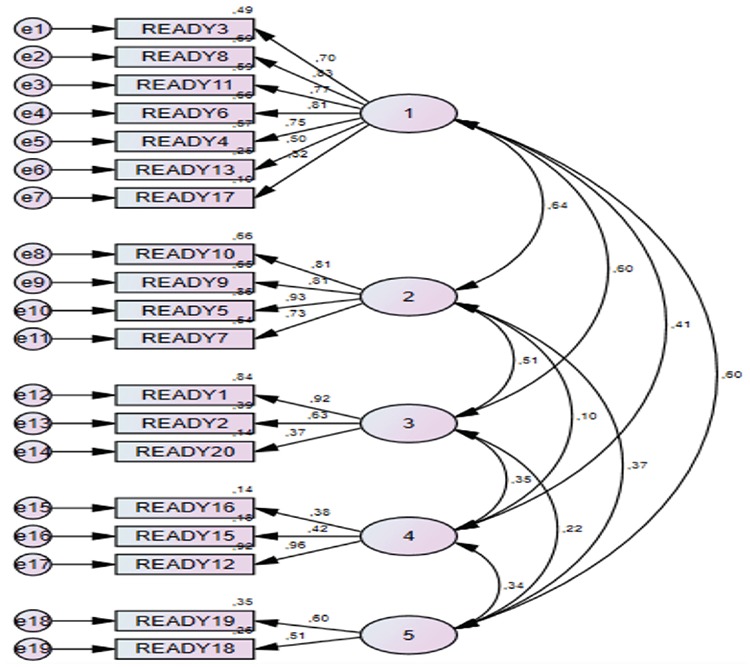
Transition readiness assessment questionnaire structural model pathdiagram.

CFA is a kind of structural equation model that is used to test whether or not the scaling model for each variable is confirmed by the data, and it has a significant value in scale adaptation studies. The scaling model correlates the observed indicators in each scale with the structure they denote, thereby giving factor loadings for each observed indicator. The model that was obtained as a result of EFA was analyzed with the same structure in the AMOS package program, and the path diagram (shown in Figure 1) was obtained. The CFA results showed that the ratio χ^2^/df of the scale was 2.49 (χ2=426.819; df=171; p=0.000). The ratios χ2/df lower than 3.0 are considered to be an indicator of good fit, although those between 4.0 and 5.0 are considered to be an acceptable fit level. The Root Mean Square Residual (RMR) index between 0 and 1 and the root mean squared error approximation (RMSEA) index lower than 0.05 are considered to be an indicator of good fit (28-30). The values and standard values of goodness-of-fit indices obtained from the scaling model in this study are shown in Table 3. With regard to the model, the ratio χ^2^/df showing overall model fit and goodness-of-fit index (GFI) and adjusted goodness-of-fit index (AGFI) values, which are certain fit indexes, show good fit. Values of comparative fit indexes, the normed fit index, relative goodness-of-fit index, incremental fit index, and RMSEA, were within acceptable limits and indicated good fit for RMR. Therefore, the quintet factor model of the TRAQ is in accordance with the sample group.

### Reliability of the Transition Readiness Assessment Questionnaire

***Item-total score correlation and internal consistency***

Table 4 shows the item-total score correlations and Cronbach’s alpha internal consistency coefficient values of questions related to the whole scale and its subdimensions (follow-up of the appointments, management of the treatment, follow-up on any health problems, communication with health care professionals, and management of daily activities). The Cronbach’s alpha internal consistency coefficient value of the whole scale was 0.88, and the values of the subdimensions ranged between 0.75 and 0.89. It was revealed that the item-total correlation of the scale items ranged between 0.40 and 0.83 after item analysis was performed in an attempt to determine the severalizing properties of the subscale items with regard to detecting what these items surveyed.

***Repeatability of the scale***

In accordance with the test-retest method, the scales were given again to 44 adolescents 3 weeks after the first application. Correlation coefficients (Pearson’s correlation) between the scores obtained in consequence of the two applications were calculated, and it was established that the test–retest correlation coefficients ranged between 0.79 and 0.93 (p<0.01).

## DISCUSSION

The aim of this study was to determine the validity and reliability of the Turkish TRAQ. Our findings indicate that the Turkish TRAQ, which was developed by Wood et al. (12) to assess the transition readiness from pediatric care to adult care of adolescents with chronic diseases, could be used for adolescents with diabetes mellitus. In adapting a scale to a different culture, the first thing to do is to translate from the original language to the target language and translate it back and assess the content validity (29,30). Validity is defined as the determination degree of a test in terms of determining the feature accurately and consistently without confusing it with different features (31). For the content validity of the scale, expert opinions were gathered, the questions of the scale were evaluated for clarity, fluency, and intelligibility, and it was determined that no changes would be made. Before the transition to adult care, a scale that was composed of 20 questions that assess the adolescents’ management of the disease, self-care abilities, and readiness to transition to adult care was replied by the sample group, and the scale was considered to be understandable and easily applicable. In testing the validity of the TRAQ, a factor analysis (EFA and CFA) and criterion validity assessment were performed. Factor analysis aims to reach scarce definable significant factors that can be defined by a large number of variables together (29,30). Variables that measure the same feature create factors by gathering in regard to their item loads. EFA resulted in a 20-item scale with five identified subscales that clarified 74% of total variance (Table 2). Moreover, it is recommended that the cumulative variance ratio of a valid scale should be at least 40% (29). According to EFA results, we could say that the Turkish TRAQ has high structural validity. The association between a statement and the factor that the statement explains is as high as the statement’s factor load. Generally, the factor load points of the items are expected to have higher values than 0.45 (32). In our study, item factor loads were evaluated via principal components analysis and varimax orthogonal rotation technique, and factor loads were calculated as 0.47-0.83.

CFA was performed, and goodness-of-fit statistics were analyzed for assessing whether the factor model obtained by EFA was fit for our sample (Table 3). Although, for model assessment, χ^2^, RMSEA, GFI, and AGFI indices were used frequently in the literature, other indices for goodness of fit are available, and there are differences between good fit and acceptable fit (32). In general, the ratios χ^2^/df lower than 3.0 are considered as indicators of good fit; nevertheless, those between 4.0 and 5.0 are considered to be an acceptable fit level. Values between 0 and 1 for the RMR and below 0.05 for RMSEA are good indicators of compliance (28,29,30). In our study, the value of the ratio χ^2^/df that shows general model fit was 2.49, while the GFI and AGFI indicated good fit. The comparative fit indices ranged within acceptable fit limits and showed that the scale model had an appropriate structure for the sample. For testing the criterion validity of the TRAQ, the self-care scale, which was developed simultaneously and equivalently with same sample group and tested for validity and reliability, was administered. When we investigated the correlation between the scores of the two scales, we established a statistically significant relationship (positively, at a level of 0.01) between the total score of the self-care ability scale and the TRAQ scores (r=0.57; p<0.01). A correlation value of ≥0.30 calculated for a criterion validity coefficient is considered to be an indicator of the validity of the test (32). According to this, we can say that the TRAQ accurately assesses the readiness of adolescents with diabetes mellitus to transition from pediatric care to adult care. Reliability of the scale was assessed via total item score correlation, internal consistency, and invariance for time methods. The total item score correlation is calculated to determine the association between the scale items and the whole scale (29,32). In the literature, items that have a total item score correlation coefficiency value higher than or equal to 0.30 are considered to have enough representative strength, whereas items that have a value lower than 0.30 are recommended to be excluded from the scale (32). In our study, we determined that no item was required to be excluded from the scale, and the total item score correlation of the scale items ranged between 0.40 and 0.83 (Table 4). In this context, we can say that the TRAQ has high distinctiveness with regards to readiness of the items.

To say that a scale has internal consistency reliability, it must be demonstrated that all subdimensions of the scale surveyed the same property. Besides, it is supposed that the higher Cronbach’s alpha coefficient values indicate that the scale items were composed of the same items (29). In our study, we calculated Cronbach’s alpha internal consistency reliability coefficient values for the whole scale and subdimensions (i.e., keeping appointment, managing medications, tracking health issues, managing daily activities, and talking with providers) (Table 4). The Cronbach’s alpha internal consistency coefficient of the whole scale was 0.88 and it ranged from 0.70-0.89 for the subdimensions. These data suggest that the Turkish TRAQ has high reliability. The Cronbach’s alpha coefficient value in the study by Wood et al. (12) was 0.94. The Cronbach’s alpha coefficient of the subdimensions of the original scale ranged between 0.67 and 0.90, and these results are similar to the alpha coefficient values in the present study (Table 4). These results indicate that the internal consistency of the Turkish TRAQ is high. One of the approaches used to assess the reliability of the measurement is the test-retest method (32). Repeatability of the scale was assessed using the test-retest method, and the test-retest reliability coefficient was calculated as 0.86 (p<0.05). When we consider the fact that the test-retest correlation coefficient, calculated to determine the invariance for the time of the scale, should be higher than 0.70, we can say that, in this study, reliability of the scale is high (32). In conclusion, the Turkish TRAQ assesses the self-management ability and HCT knowledge of AYA with chronic diseases. Therefore, it assesses the transition readiness of the individual. It is also considered to be a guide for health care professionals for detecting the educational fields that is required to gain self-management and self-care abilities. The TRAQ, which is recommended by the American Academy of Pediatrics for application in adolescents from 14 years of age upward regularly, every 6-12 months, is also a valid and reliable survey for Turkish adolescents with diabetes mellitus. For adolescents with other chronic diseases, the adaptation to the Turkish language as well as validity and reliability studies of the scale are still in progress.

## Figures and Tables

**Table 1:**
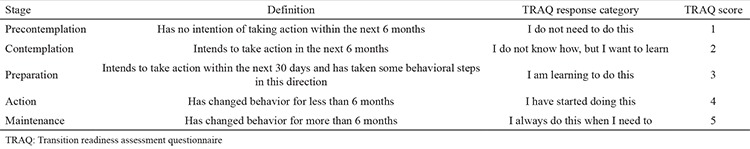
Stages of change model for the TRAQ development (18,25)

**Table 2:**
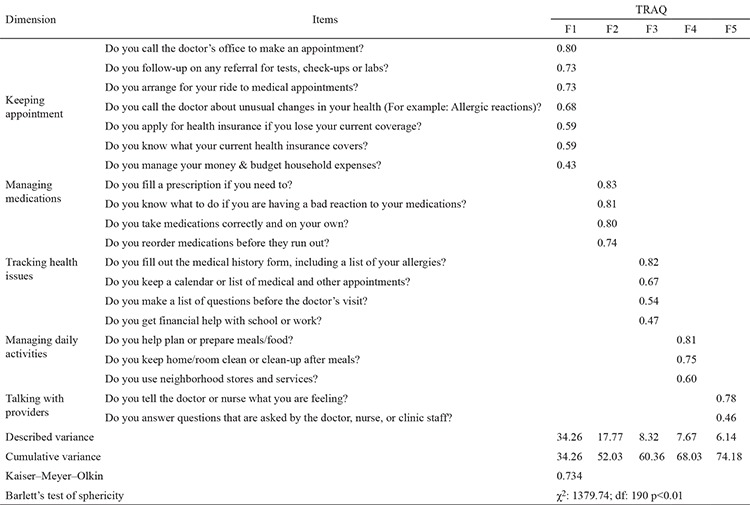
Factor loadings of the items

**Table 3:**
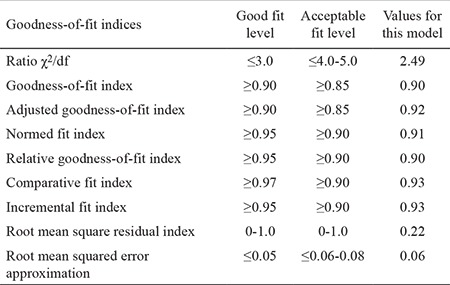
Goodness-of-fit indices for the model

**Table 4:**
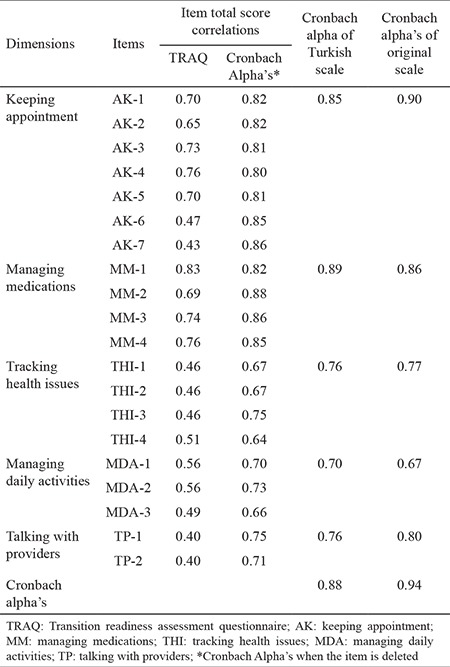
Item-total score correlations and cronbach alpha’s of the TRAQ

## References

[1] Sheehan A, While A, Coyne I (2015). The experiences and impact of transition from child to adult healthcare services for young people with Type 1 diabetes: a systematic review. Diabet Med.

[2] Lotstein DS, Seid M, Klingensmith G, Case D, Lawrence JM, Pihoker C, et al (2013). Transition from pediatric to adult care for youth diagnosed with type 1 diabetes in adolescence. Pediatrics.

[3] Committee On Pediatric Aids (2013). Transitioning HIV-infected youth into adult health care. Pediatrics.

[4] Prior M, McManus M, White P, Davidson L (2014). Measuring the "triple aim" in transition care: a systematic review. Pediatrics.

[5] DeBaun MR, Telfair J (2012). Transition and sickle cell disease. Pediatrics.

[6] Crowley R, Wolfe I, Lock K, McKee M (2011). Improving the transition between paediatric and adult healthcare: a systematic review. Arch Dis Child.

[7] Aldiss S, Ellis J, Cass H, Pettigrew T, Rose L, Gibson F (2015). Transition From Child to Adult Care 'It's Not a One-Off Event': Development of Benchmarks to Improve the Experience. J Pediatr Nurs.

[8] Ferris ME, Harward DH, Bickford K, Layton JB, Ferris MT, Hogan SL, et al (2012). A clinical tool to measure the components of health-care transition from pediatric care to adult care: the UNC TR(x)ANSITION scale. Ren Fail.

[9] Ferris ME, Mahan JD (2009). Pediatric chronic kidney disease and the process of health care transition. Semin Nephrol.

[10] Bryant R, Young A, Cesario S, Binder B (2011). Transition of chronically ill youth to adult health care: experience of youth with hemoglobinopathy. J Pediatr Health Care.

[11] Cadario F, Prodam F, Bellone S, Trada M, Binotti M, Allochis G, et al (2009). Transition process of patients with type 1 diabetes (T1DM) from paediatric to the adult health care service: a hospital-based approach. Clin Endocrinol (Oxf).

[12] Wood DL, Sawicki GS, Miller MD, Smotherman C, Lukens-Bull K, Livingood WC, et al (2014). The Transition Readiness Assessment Questionnaire (TRAQ): its factor structure, reliability, and validity. Acad Pediatr.

[13] Blinder MA, Vekeman F, Sasane M, Trahey A, Paley C, Duh MS (2013). Age-related treatment patterns in sickle cell disease patients and the associated sickle cell complications and healthcare costs. Pediatr Blood Cancer.

[14] Heery E, Sheehan AM, While AE, Coyne I (2015). Experiences and Outcomes of Transition from Pediatric to Adult Health Care Services for Young People with Congenital Heart Disease: A Systematic Review. Congenit Heart Dis.

[15] Wodrich DL, Hasan K, Parent KB (2011). Type 1 diabetes mellitus and school: a review. Pediatr Diabetes.

[16] Colver A, Longwell S (2013). New understanding of adolescent brain development: relevance to transitional healthcare for young people with long term conditions. Arch Dis Child.

[17] Sawicki GS, Lukens-Bull K, Yin X, Demars N, Huang IC, Livingood W, et al (2011). Measuring the transition readiness of youth with special healthcare needs: validation of the TRAQ--Transition Readiness Assessment Questionnaire. J Pediatr Psychol.

[18] Kapellen TM, Kiess W (2015). Transition of adolescents and young adults with endocrine diseases to adult health care. Best Pract Res Clin Endocrinol Metab.

[19] Cooley WC, Sagerman PJ, American Academy of Pediatrics, American Academy of Family Physicians, American College of Physicians, Transitions Clinical Report Authoring Group (2011). Supporting the health care transition from adolescence to adulthood in the medical home. Pediatrics.

[20] Hersh AO, Pang S, Curran ML, Milojevic DS, von E (2009). The challenges of transferring chronic illness patients to adult care: reflections from pediatric and adult rheumatology at a US academic center. Pediatr Rheumatol Online J.

[21] van Staa A, van der Stege HA, Jedeloo S, Moll HA, Hilberink SR (2011). Readiness to transfer to adult care of adolescents with chronic conditions: exploration of associated factors. J Adolesc Health.

[22] Tuchman LK, Schwartz LA, Sawicki GS, Britto MT (2010). Cystic fibrosis and transition to adult medical care. Pediatrics.

[23] Hayran M (2011). Basic statistics for medical researches. 1st ed Ankara. Omega publication.

[24] Prochaska JO, DiClemente CC (2005). The transtheoretical approach. Handbook of psychotherapy integration..

[25] Nahcivan N, Tuncel N (1999). Self care agency in healthy adolescents and effect of family. Florence Nightingale HD.

[26] Nahcivan NO (2004). A Turkish language equivalence of the Exercise of Self-Care Agency Scale. West J Nurs Res.

[27] Byrne BM (2013). Structural equation modeling with AMOS: Basic concepts, applications, and programming. Routledge.

[28] Meydan CH, Şeşen H (2011). Structural equation modeling with AMOS. Detay publication.

[29] Şimşek ÖF (2007). Structural equation modeling: Basic principles and LISREL practices. Ankara: Ekinoks.

[30] Kalaycı Ş (2006). Multivariate statistical techniques. Asil publication.

[31] Büyüköztürk Ş, Akgün ÖE, Kahveci Ö, Demirel F (2004). The Validity and Reliability Study of the Turkish Version of the Motivated Strategies for Learning Questionnaire. KUYEB.

[32] Akgül A (2005). Statistical analysis in medical research: SPSS practices. Council of Higher Education Press.

